# Core Self-Evaluations Increases Among Chinese Employees: A Cross-Temporal Meta-Analysis, 2010–2019

**DOI:** 10.3389/fpsyg.2021.770249

**Published:** 2022-02-07

**Authors:** Xinqi Lin, Yuxiang Luan, Guolong Zhao, Teng Zhao, He Ding

**Affiliations:** ^1^School of Labor and Human Resources, Renmin University of China, Beijing, China; ^2^Department of Psychological Sciences, Auburn University, Auburn, AL, United States; ^3^School of Economics and Management, North China Electric Power University, Beijing, China

**Keywords:** core self-evaluations, cross-temporal meta-analysis, Chinese employees, CSES, meta-analysis

## Abstract

The purpose of this paper is to investigate the changes in core self-evaluation (CSE) scores among Chinese employees during 2010–2019. We conducted a cross-temporal meta-analysis including 50 studies (17,400 Chinese employees) to evaluate the relationship between the year of data collection and levels of CSE. We found that correlations between levels of CSE and year of data collection were strong and positive (*r* > 0.500). Regression results showed that the year of data collection could predict the CSE score when the mean sample age and sex ratio (%female) were controlled. In addition, CSE scores were positively related to GDP per capita and negatively related to the unemployment rate.

## Introduction

The economy of China has proliferated in the past 40 years because of the Reform and Opening-up Policy, influencing every single individual in this country. On the one hand, the physical conditions of Chinese people changed quickly. For example, Chinese people become healthier and tend to have higher life expectancies. On the other hand, the psychological aspects of Chinese people changed. For instance, previous cross-temporal meta-analyses showed that personality (Peng and Luo, 2021; Su and Liu, 2021), loneliness (Yan et al., 2014), anxiety (Xin et al., 2010), and mental health (Silin et al., 2015) among Chinese people have changed over time.

Personality is determined by gene expression and may be influenced by variation in environments (Roberts and Jackson, 2008). Since Chinese employees face the great growth of the economy directly, the quick changes of the economy and society may influence their personality. Core self-evaluations (CSE) is a higher-order personality, defined as “fundamental premises that individuals hold about themselves and their functioning in the world” (Judge et al., 1998), which have received growing academic attention over recent years.

Although previous studies researched the changes of sub-traits of CSE (e.g., locus of control, Twenge et al., 2004; self-esteem, Gentile et al., 2010), there are a lack of studies systematically researching the changes of CSE scores among Chinese people. Such research is important to theory because it would (1) increase our knowledge about CSE, (2) promote our understanding of the changes of CSE and its links with economic growth, and (3) increase our knowledge about Chinese employees' psychological aspects. Our study is also important for practice. Since CSE is an important personality trait that could predict job performance and job satisfaction (Judge and Bono, 2001; Judge and Kammeyer-Mueller, 2011), such a study would help managers to better understand the changes in their employees' psychological state and provide better management and motivation to them.

In this research, we investigated whether Chinese employees have a more positive core self-evaluation, namely, whether the CSE scores among Chinese employees increased in the past few years. In addition, we want to know how the potential relationship between economic growth and CSE scores changes over time.

## Hypotheses Development

### CSE and Its Measurement

To begin with, we briefly review CSE. CSE is a higher-order trait that includes four well-known sub-traits: self-esteem, locus of control, generalized self-efficacy, and emotional stability (Judge and Bono, 2001; Judge and Kammeyer-Mueller, 2011). First, self-esteem represents the affective, or evaluative component of the self-concept, signifying how people feel about themselves (Leary and Baumeister, 2000). Second, locus of control is categorized into internals and externals. Internals believe they are in control of their destinies and thus they have an internal locus of control; on the contrary, externals believe that luck and powerful others determine their fate and thus they have an external locus of control (Twenge et al., 2004). Third, generalized self-efficacy represents one's estimate of one's fundamental ability to cope, perform, and potential to be successful (Judge and Bono, 2001). Fourth, emotional stability refers to the extent to which a person is calm and poised (Peeters et al., 2006). People high in positive CSE usually have high self-esteem, generalized self-efficacy, emotional stability, and internal locus of control. Namely, they see themselves positively across various situations and approach the world in a confident, self-assured manner (Judge and Kammeyer-Mueller, 2011).

As a cross-temporal meta-analysis, it is necessary to compare the scores of a construct. Although including four well-known sub-traits, CSE is not a multidimensional aggregate construct. If so, its four sub-traits should not relate to each other or have relatively low correlations. However, a meta-analysis showed that correlations among them were high (r > 0.500) (Judge and Bono, 2001). Moreover, Judge et al. (2003) argued that CSE influences four sub-traits and developed a 12-items Core Self-Evaluation Scale (CSES). Considering the high reliability and validity of CSES, we are drawing on CSES rather than another measurement of CSE in this study.

### CSE Changes

Since there is a lack of research which studies the change of CSE levels, we reviewed studies that researched four sub-traits of CSE (see Table 1). To begin with, for the locus of control, Twenge et al. (2004) showed that young Americans increasingly believe their lives are controlled by outside forces rather than their efforts during 1960–2002, namely, an increasing number of young Americans were becoming externals. Next, for self-esteem, many studies were researched, and different conclusions remained. For example, Twenge and Campbell (2001) found that American children's self-esteem scores decreased from 1965 to 1979 and increased from 1980 to 1993. Besides, Gentile et al. (2010), Liu and Xin (2015), and Hamamura and Septarini (2017) studied self-esteem changes among different samples (see Table 1). Last but not least, for emotional stability, Peng and Luo (2021) found that neuroticism among Chinese college students increased from 2001 to 2016.

**Table 1:** Previous cross-temporal meta-analyses of CSE.

**References**	**Variable**	**Country**	**Sample**	**Year**	**Results**	**Correlation**
Twenge et al. (2004)	Locus of control	US	College students and children	1960–2002	Locus of control scores became substantially more external	*r* = 0.700
Twenge and Campbell (2001)	Self-esteem	US	children	1965–1993	self-esteem decreasing from 1965 to 1979 and increasing from 1980 to 1993	-
Hamamura and Septarini (2017)	Self-esteem	Australia	high school students, university students, and community participants	1978–2014	Self-esteem did not change	-
Liu and Xin (2015)	Self-esteem	China	ADOLESCENTS	1996–2009	Self-esteem decreasing from 1996 to 2009	*r* = −0.390
Gentile et al. (2010)	Self-esteem	US	middle school, high school, and college students	1988–2008	Self-esteem increasing from 1988 to 2008	*r* = 0.770,0.630 and 0.040 (for middle school, high school, and college students)
Peng and Luo (2021)	Neuroticism	China	College students	2001–2016	Neuroticism increasing from 2001 to 2016	*r* = 0.280

Drawing on the review and table above, no study directly researched how CSE scores change and examined employee samples in China. However, the tremendous economic growth in China may influence employees directly and profoundly, especially in psychological aspects. Unfortunately, we know little about it. To address these gaps, we would like to study CSE score changes among Chinese employees. Therefore, we propose the first question:

Research Question 1: Did CSE scores among Chinese employees change over time from 2010 to 2019?

### Economic Growth and CSE Score Changes Over Time

Economic changes may influence personality. We expect that the CSE scores among Chinese employees would increase in response to economic indexes during 2010–2019. First, Greenfield (2009) proposed a theory arguing that economic change may influence culture and human development; specifically, economic growth increases individualism (focus on the self and personal possessions), while economic recession decreases individualism. Previous studies found narcissism, which is characterized by an inflated sense of the self and self-entitlement (Gnambs and Appel, 2018) increased between 1982 and 2009 and declined after the Great Recession among U.S. college students (Twenge et al., 2021). Narcissism was moderately related to self-esteem (Brown and Zeigler-Hill, 2004), a sub-trait of CSE, and related to CSE directly (Rode et al., 2012). Second, CSE reflects the overall evaluations of self. People high in CSE would be confident and believe they could control their fate (Judge and Kammeyer-Mueller, 2011). When the economy grows quickly, people would feel more confident because they have more opportunity to find success in the workplace (Twenge et al., 2021). However, when the economic crisis arrives, people may suffer more from the bad economic environment, facing more stress and becoming less confident. Third, when a survey study had two samples from different countries, we found that CSE scores were higher in the country with a higher economic level. For instance, we found that CSE scores in the US were higher than in China (Rode et al., 2012), India (Prather-Kinsey et al., 2018), Korea (Flynn et al., 2015), and Turkey (Costigan et al., 2016). The US has better economic conditions, which may reveal that people from an area with better economic conditions would have higher CSE scores. If so, when there is economic growth in a country, its CSE scores may also increase. Thus, considering the influence of economic growth, the CSE scores among Chinese employees may increase during 2010–2019. We studied the trends during 2010–2019 because few studies tackled the personality of CSE among Chinese employees before 2010.

Since we believe that better economic conditions may have higher CSE scores, we need to find some critical economic indexes that could reflect the economic conditions of a country. First, Gross Domestic Product (GDP), which is defined as the “monetary, market value of all final goods and services produced in a country over a period of a year” (Bergh, 2009), is considered. GDP is one of the most important economic indexes in Macroeconomics. GDP per capita was found to be positively related to a critical index of well-being, such as social welfare (Beckerman, 2002), education (Islam et al., 2007), and life expectancy (Ebenstein et al., 2015). GDP per capita change could reflect the overall growth of its economy, which in turn might positively relate to the CSE scores. Second, the unemployment rate is also an important index. Economic growth leads to an increase in demand. To maintain the balance between market demand and supply, supply also needs to increase. Therefore, production will increase. The increase of production requires capital and labor to be invested (the most basic factor of production), so employment will increase. However, economic recession decreases the demand, which in turn, increases unemployment. The unemployment rate was found to be related to education (Wolbers, 2000), and education has an impact on personality (Dahmann and Anger, 2014). Besides, the unemployment rate was related to narcissism (Twenge et al., 2021) and self-esteem (Twenge and Campbell, 2001) in previous studies. In sum, as an important economic index, the unemployment rate may negatively relate to the CSE scores. Therefore, we put forward the following hypotheses:

Hypothesis 1: CSE scores are positively related to GDP per capita among Chinese employees.

Hypothesis 2: CSE scores are negatively related to the unemployment rate among Chinese employees.

## Methods

The purpose of the current study is to investigate the changes in CSE levels among Chinese employees during 2010–2019 and the relationship between economic indexes and the levels of CSE. We conducted a cross-temporal meta-analysis to accomplish our research goal. Drawing on CSES (Judge et al., 2003), we compared the level of CSE among Chinese employees in different years. Specifically, we collected the scores of CSES among Chinese employees during 2010–2019. Next, we weighed the scores of CSES by sample size and inverse variance. Then, we analyzed the relationship between the scores of CSES, GDP per capita, unemployment rate, and year of data collection. Finally, we researched the potential moderating effects of sample age and sex ratio on the relationship between CSE scores and year.

### Literature Search

We searched the studies that might include the level of CSE among Chinese employees using the following databases: PsycINFO, EBSCO, Web of Science, Google Scholar, and China National Knowledge Infrastructure (CNKI). We determined keywords according to previous meta-analyses about CSE (Judge and Bono, 2001; Chang et al., 2011). We used a group of keywords referring to CSE: core self-evaluations, CSE, and CSES. The period of each search spanned publications between January 2010 and June 2021. We searched English and Chinese studies using different strategies. To search papers written in English, we used the additional keywords “Chinese” to locate studies that include Chinese samples (Peng and Luo, 2021) in the following database: PsycINFO, Web of Science, Google Scholar. To search papers writing in Chinese, we searched in CNKI, the most widely-used database of scholarly papers in China. To control the quality of Chinese papers, we only searched papers collected in the Chinese Social Sciences Citation Index (CSSCI).

### Inclusion and Exclusion Criteria

**Figure 1:**
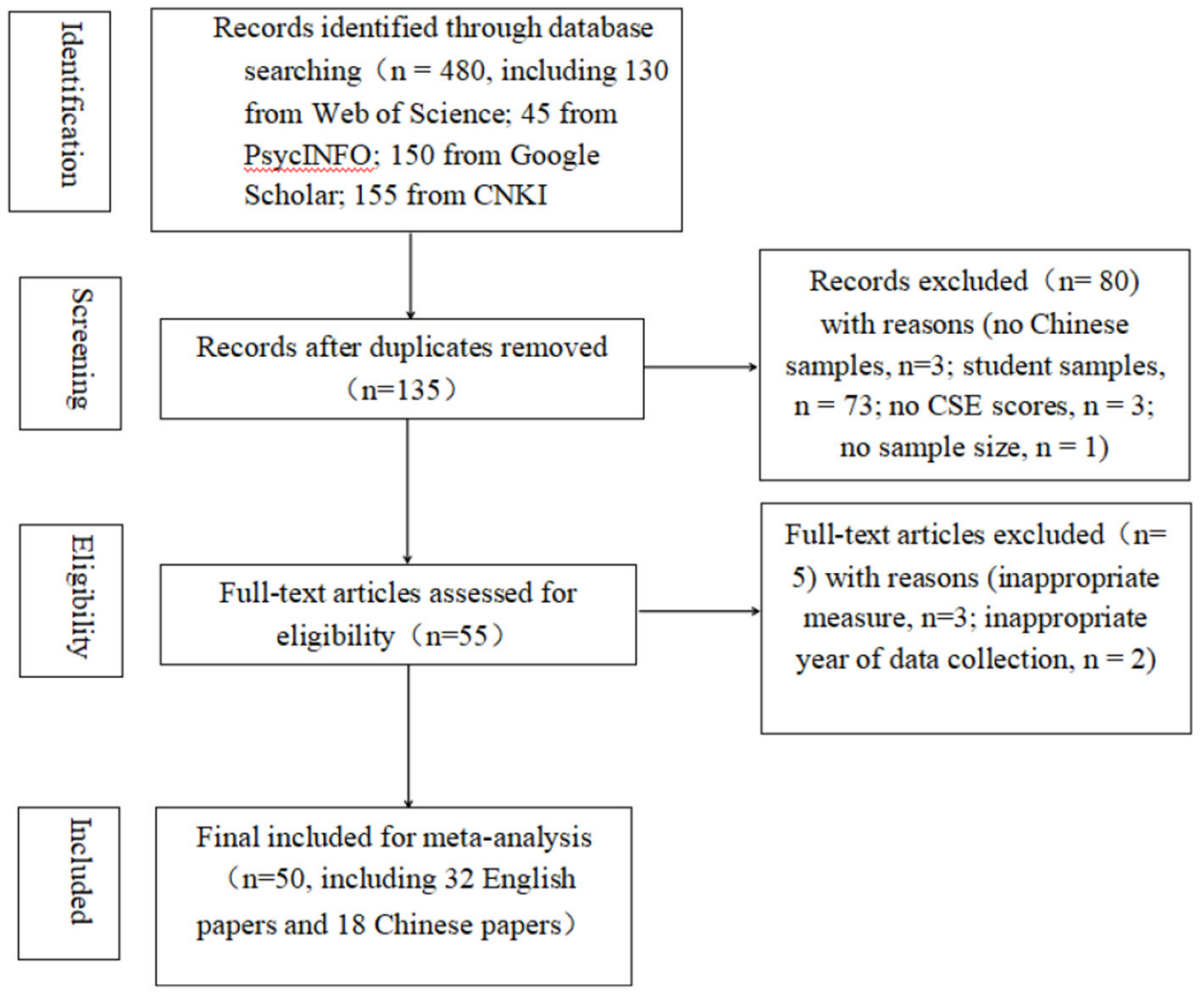
PRISMA flowchart.

Studies were included in this meta-analysis based on the following criteria: (a) CSE scores must be reported. Total score (all items added together) or mean score (all items added together divided by the number of items) are both acceptable because they could be transformed into each other (Curran and Hill, 2019); (b) samples are Chinese employees rather than students or adolescents; (c) studies must report sample size, and (d) the study included a sample that was not replicated elsewhere (studies/data sets were included only once). We also excluded studies based on the following exclusion criteria: (a) CSE scores were measured not using CSES, and (b) year of data collections were not from 2010 to 2019. A PRISMA flowchart was provided (see Figure 1).

### Coding Procedure

Two researchers coded information independently. To get the year of data collection, we adhered to the following procedure: (1) if the year was reported in the paper, we coded it directly; (2) if the year could not be found in the paper, we coded the year as two years prior to publication (Wegman et al., 2016). To code the scores of CSE, we transformed raw scores into a 100 mark system scores. For example, if a study used a 7-point scale and reported a mean score, we divided the mean score by 7 and multiplied it by 100; if a study used a 7-point scale and reported total score, we divided the total score by 84 and multiplied it by 100 (CSES has 12-items so the total score equals to 84). In addition, standard deviation (SD), bibliographic references (authors' name), and sample descriptions (sample size, the sample mean age, and sample sex ratio (%female) were coded. The average intercoder percentage of agreement across the study variables was 98%. After coding all necessary information, discussions were conducted until a consensus was reached. Finally, we had 50 scores among Chinese employees from 50 independent studies (32 papers were written in English and 18 were written in Chinese). The coding information could be found in Supplementary Table S1.

### Data Analysis

As a cross-temporal meta-analysis, we need to examine the change in scores on psychological measures over time. Previous studies used sample size or inverse variance as the weighting factor to weight the score of scales (Twenge and Campbell, 2001; Twenge et al., 2004; Wegman et al., 2016). It is quite hard to say which weighting method is better, so we used two weighting methods to get results in the current meta-analysis. When using inverse variance as the weighting factor, the weighting term (ω) equals to sample size (n) divided by the square of SD (ω = n /(SD)^2^) (Wegman et al., 2016). For these studies that did not report SD, we used the average SD (Wegman et al., 2016). We used two methods to weight CSE scores and calculate the correlations between CSE scores weight by sample size or inverse variance, year of data collection, GDP per capita, and unemployment rate (see Table 2). GDP per capita and the unemployment rate were obtained from the National Bureau of Statistics of China and shown in Supplementary Table S2.

**Table 2:** Means, standard deviations, and correlations between variable.

**Mean (SD)**		**Year**	**MCSE1**	**MCSE2**	**Unemployment**	**GDP per capita**
2014.5 (3.03)	Year	1	0.589^†^	0.577†	−0.759*	0.995**
66.1 (4.44)	MCSE1	0.589^†^	1	0.952**	−0.557^†^	0.560^†^
66.6 (4.75)	MCSE2	0.577^†^	0.952**	1	−0.707*	0.565^†^
3.99 (0.15)	Unemployment	−0.759*	−0.557^†^	−0.707*	1	−0.784**
49642.4 (12791.87)	GDP per capita	0.995**	0.560^†^	0.565^†^	−0.784**	1

*Year, year of data collection; MCSE1, mean CSE scores weighted by sample size; MCSE2, mean CSE scores weighted by inverse variance (ω)*;

** *p < 0.01*;

* *p < 0.05*;

† *p < 0.1*.

**Figure 2:**
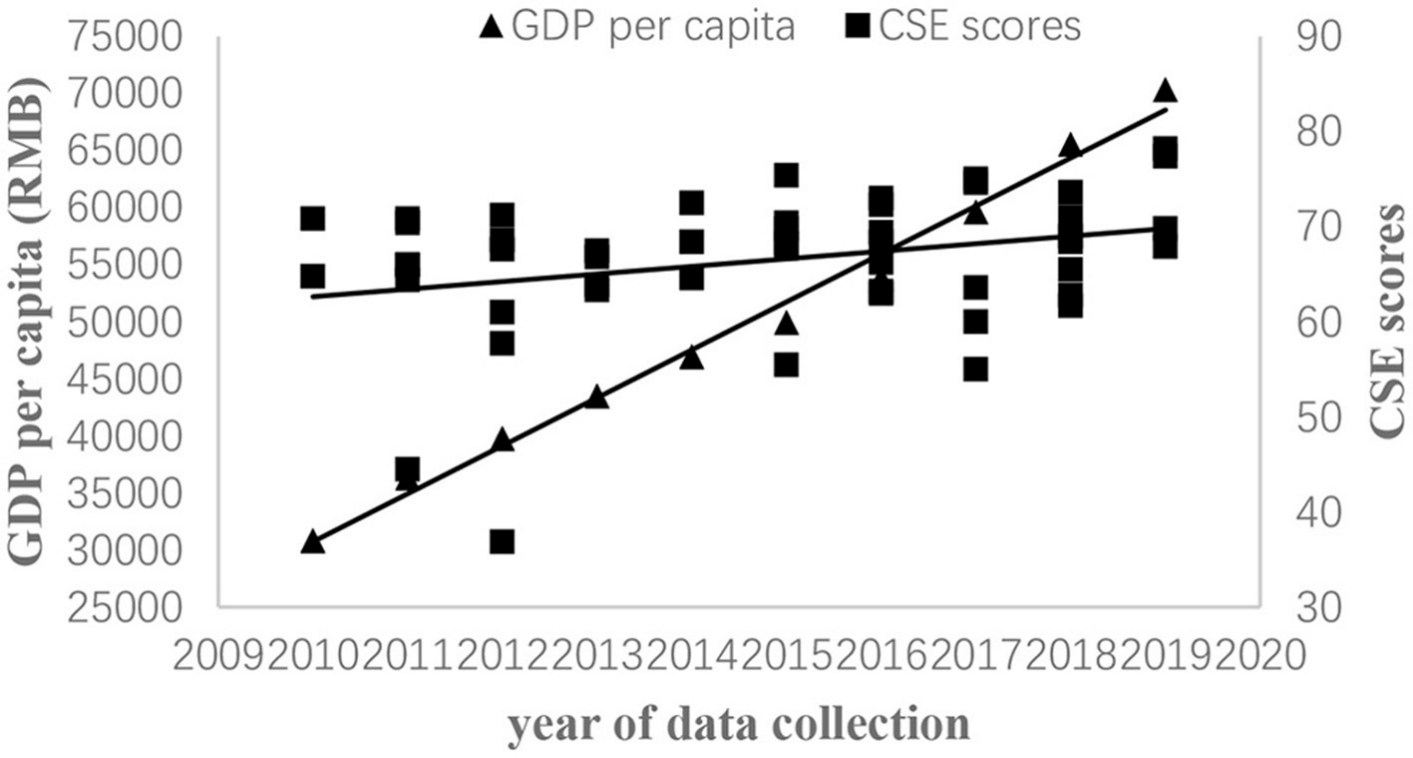
Scatter plot of GDP per capita and CSE scores among Chinese employees, 2010–2019.

**Figure 3:**
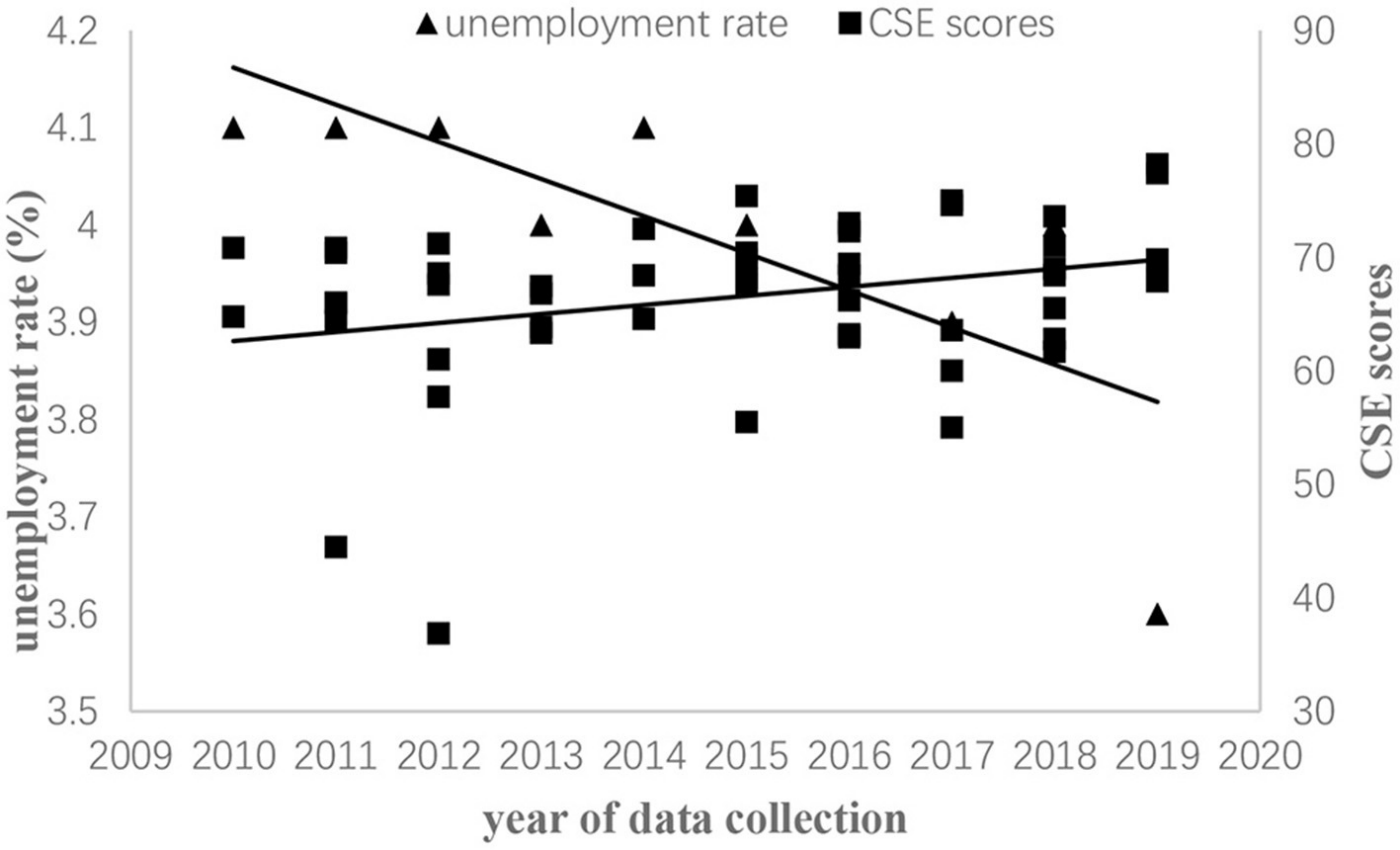
Scatter plot of the unemployment rate and CSE scores among Chinese employees, 2010–2019.

We drew two scatter plots to help readers better understand the relationship between GDP per capita, unemployment rate, and CSE score changes over time (see Figures 2, 3).

We conducted weighted linear regression to analyze the relationships between the CSE scores and the year of data collection. The year of data collection was used as the predictor, with the CSE scores as the outcome. Besides, we considered the potential moderating effects of the mean sample age and sex ratio (%female). For age, Twenge and Campbell (2001) found that self-esteem scores among young Americans increased with age. For sex, Zuckerman et al. (2016) found weak but significant sex differences in self-esteem. As a sub-trait of CSE, self-esteem could reflect CSE to some extent. Thus, we considered their potential moderating effects of age and sample sex. We used mean age and sex ratio as moderators in our regression equations. For these studies did not report mean sample age and sex ratio of the sample (% female), the average mean sample age and sex ratio of sample (% female) were used. Mean sample age, sex ratio, and their interactions with year were included in a multiple weighted linear regression model. The results were shown in Table 3.

**Table 3:** Regression results of CSE scores and year of data collection.

**Model**	**Variable**	**B**	**SE B**	**β**	**t**	**R^**2**^**	**ΔR^**2**^**
Model 1	Year	1.100	0.413	0.359	2.663*	0.129	
Model 2	Age	−0.008	0.335	−0.004	−0.024	0.159	0.030
	Year	1.100	0.425	0.359	2.586*		
	Age*year	−0.003	0.133	−0.003	−0.022		
Model 3	Sex	−0.008	0.063	−0.021	−0.135	0.143	0.014
	Year	1.064	0.421	0.347	2.529*		
	Sex*year	−0.023	0.027	−0.131	−0.826		
Model 4	Year	0.796	0.370	0.297	2.154*	0.088	
Model 5	Age	−0.141	0.321	−0.062	−0.440	0.172	0.084
	Year	1.157	0.402	0.399	2.881**		
	Age*year	−0.050	0.112	−0.064	−0.447		
Model 6	Sex	0.021	0.063	0.055	0.341	0.170	0.082
	Year	1.172	0.410	0.404	2.857**		
	Sex*year	−0.001	0.028	−0.008	−0.050		

*From model 1 to 3, CSE scores were weighted by sample size; from model 4 to 6, CSE scores were weighted by inverse variance; B, unstandardized coefficient; β, standardized coefficient; year, year of data collection; age, mean sample age; sex, sex ratio of sample (% female)*;

** *p < 0.01*;

* *p < 0.05*.

## Results

CSE scores among Chinese employees appeared to increase from 2010 to 2019 (see Figures 2, 3). From Table 2, CSE scores are positively related to the year. Two weighting methods revealed similar results [*r* = 0.589 (weighting by sample size) and *r* = 0.577 (weighting by inverse variance)]. Besides, from Table 3, the year could predict CSE scores when considering the influence of mean sample age and sex ratio (% female) and using different weight factors. Specifically, first, we found that year could predict CSE scores whether CSE scores were weighted by sample size (β = 0.359, *p* < 0.050 ) or inverse variance (β = 0.297, *p* < 0.050) (see model 1 and model 4, Table 3). Second, the interactions with mean sample age were not significant (β = −0.003, *p* > 0.100, see model 2; β = −0.064, *p* > 0.100, see model 5), mean sample age did not have a significant moderating effect. Similarly, the sex ratio of the sample (% female) also did not have a significant moderating effect (see model 3 and model 6). In sum, results showed that CSE scores among Chinese employees increased from 2010 to 2019. Age and sex ratio did not have significant moderating effects. In sum, our results answer the first research question that CSE scores among Chinese people increased from 2010 to 2019.

Next, we examined the relationships between CSE scores and two economic indexes. First, for GDP per capita, drawing on Figure 2, we found that both CSE scores and GDP per capita increased. Moreover, mean CSE scores positively related to GDP per capita whether CSE scores were weighted by sample size (*r* = 0.560) or inverse variance (*r* = 0.565) (see Table 2). Second, for the unemployment rate, based on Figure 3, we found that CSE scores increased while the unemployment rate decreased. Besides, unemployment rate was negatively related to mean CSE scores whether CSE scores were weighted by sample size (*r* = −0.557) or inverse variance (*r* = −0.707) (see Table 2). In sum, two economic indicators revealed that CSE scores increased when the economy grew. To sum up, H1 and H2 were supported.

## Discussion

### Theoretical Implications

Our study is the first one to investigate the changes in CSE scores drawing on cross-temporal meta-analysis. China has gained so much economic achievement over the past 40 years. The tremendous economic growth influences every person and their psychological aspects in this country. However, few studies focus on the changes in personality in China. We made a cross-temporal meta-analysis of 50 samples of Chinese employees and found that CSE scores increased from 2010 to 2019. According to the definition of CSE (Chang et al., 2011; Judge and Kammeyer-Mueller, 2011), our results showed that Chinese employees saw themselves more and more positively across a variety of situations and approached the world in a more and more confident, self-assured manner during 2010 to 2019. Our research contributes to the knowledge of understanding the changes in psychological aspects among Chinese people.

Age and sex ratio did not significantly influence the relationship between CSE scores and year of data collection. For age, a possible explanation is CSE scores may change little among adults. For example, a longitudinal study showed mean CSE scores among adults changed little (Wu and Griffin, 2012). Besides, Roberts et al. (2006) also found that emotional stability, a sub-trait of CSE, changed very little in adulthood. For sex, the results surprised us because men seem to have higher self-esteem, self-confidence, and more positive self-views (Lenney et al., 1983). Although the moderating effects of sex ratio were not significant (p > 0.050), the interactive items between year and sex ratio (% female) were negative (see model 3 and model 6, Table 3), which revealed that samples with more males might have higher CSE scores. CSE drew so much academic attention over several decades, however, we found few studies which paid attention to sex differences. We encourage more studies to research the sex differences in CSE.

We also investigate the relationships between CSE scores and two important economic indexes. First, GDP per capita was found positively related to CSE scores. There is no doubt that GDP is the most important economic index in the world. The increasing of GDP means the overall economic conditions in a country are becoming better. Second, the unemployment rate was found negatively related to CSE scores. When the economy increases, the unemployment rate usually decreases. Two economic indexes revealed that better economic conditions might make people have more positive self-views. Namely, their CSE scores were increasing. Twenge et al. (2021) found that unemployment was negatively related to Narcissistic Personality Inventory scores, which means better economic conditions may be positively related to narcissism. Actually, both narcissism and CSE reflect the focus on self or ego, economic growth may make people focus on the self and personal possessions (Greenfield, 2009).

The current study only revealed the correlations between CSE scores and economic indexes rather than causal relationships. In fact, reverse causality may exist, namely, higher CSE scores may influence economic conditions. Specifically, CSE was believed to be a reliable personality to predict employee performance (Judge et al., 2003; Chang et al., 2011). When CSE scores among employees increase, employees may have higher performance and be more productive, increasing GDP per capita. In sum, drawing on the research design of the current study, we could only find the correlations between CSE scores and economic indexes and not exclude the reverse causality between them.

### Practical Implications

Our meta-analysis also has some practical implications. First, our study helps Chinese managers to know the link between economic growth and CSE changes, thereby they could use more accurate practices to motivate their employees. Second, our research provides evidence for Chinese economic policymakers that it is meaningful to maintain economic growth by using suitable macroeconomic policies because it can promote the positive changes in the people's personality traits. Considering that the CSE reflects the overall evaluations of the world (Judge et al., 1998) when a person's CSE is more positive, they are more likely to see the world from a positive perspective and increase a series of positive consequences (e.g., self-confidence, work performance, and overall life satisfaction). In other words, by keeping economic growth, the government may offer their people more confidence and happiness.

### Limitations

Some limitations of our study should be mentioned. First, as a cross-temporal meta-analysis, the CSE scores may not be randomly sampled. Future studies could sample randomly and use longitudinal data to research CSE changes over time. Second, we only studied the overall CSE rather than four sub-traits of CSE among Chinese employees. We encourage future studies to focus on four sub-traits of CSE. Third, we only studied two important economic indexes. Future studies could consider more complex and reliable indexes.

## Conclusions

The economy of China has experienced rapid development for more than 40 years, which has had huge impacts on the physical and psychological states of every Chinese employee. We quantitatively analyzed the changes in CSE of Chinese employees drawing on cross-temporal meta-analysis technology. We collected evidence from 50 studies (17,400 Chinese employees) and found that CSE scores among Chinese employees increased from 2010 to 2019. We used two weighted methods and considered the potential moderating effects of the sample mean age and sample sex (% female); the results remain significant. Besides, we found CSE scores among Chinese employees were positively related to GDP per capita and negatively related to the unemployment rate. Our study contributes to CSE literature and helps to understand the changes in Chinese the personalities of Chinese people. Moreover, our study provides evidence to managers and policymakers in China.

## Data Availability Statement

The original contributions presented in the study are included in the article/Supplementary Material, further inquiries can be directed to the corresponding author/s.

## Author Contributions

XL: idea and introduction. YL: method, results, and coding. GZ: discussing and coding. TZ: coding and revising language. HD: method, coding, and revising language. All authors contributed to the article and approved the submitted version.

## Conflict of Interest

The authors declare that the research was conducted in the absence of any commercial or financial relationships that could be construed as a potential conflict of interest.

## Publisher's Note

All claims expressed in this article are solely those of the authors and do not necessarily represent those of their affiliated organizations, or those of the publisher, the editors and the reviewers. Any product that may be evaluated in this article, or claim that may be made by its manufacturer, is not guaranteed or endorsed by the publisher.
