# Supplementary material for: Core Self-Evaluations Increases Among Chinese Employees: A Cross-Temporal Meta-Analysis, 2010–2019
Source: Front Psychol. 2022 Feb 7;12:770249. doi: 10.3389/fpsyg.2021.770249 (PMC8858941; doi:10.3389/fpsyg.2021.770249)
Supplement: Supplementary file 1 [file Table_1.doc]

Table S1 Coding Information

| NO. | name | Year | N | CSE Score | SD | age | sex |
| --- | --- | --- | --- | --- | --- | --- | --- |
|  | | | | | | | |
| 1 | Joseph C. Rode1 | 2012 | 269 | 61.00 | 0.49 | 33.96 | 58 |
| 2 | Muhammad Usman | 2018 | 260 | 73.60 | 0.89 | ？ | 46.5 |
| 3 | Zibin Song | 2013 | 161 | 63.86 | 0.74 | ？ | ？ |
| 4 | Hui-Hsien Hsieh | 2016 | 346 | 68.40 | 0.46 | ？ | 67.3 |
| 5 | Yiling Hu | 2016 | 187 | 73.00 | 0.48 | 37.89 | 48.6 |
| 6 | Zhen Wang | 2018 | 223 | 65.50 | 0.54 | 38.5 | 75 |
| 7 | He Ding | 2018 | 406 | 69.00 | 0.52 | 34.81 | 54.2 |
| 8 | Samuel Aryee | 2015 | 276 | 70.40 | 0.43 | 34.42 | 49 |
| 9 | Yen-Chun Peng | 2014 | 262 | 72.50 | 0.77 | 50.55 | 48.2 |
| 10 | Yu Ha Cheung | 2016 | 154 | 63.20 | 0.44 | 29.35 | 58 |
| 11 | Jia Hu | 2011 | 150 | 70.83 | 0.5 | 32.56 | ? |
| 12 | Feng Kong | 2013 | 310 | 63.33 | ? | ? | ? |
| 13 | JIAN LIANG1 | 2011 | 169 | 70.40 | 0.44 | ? | 26 |
| 14 | Thomas | 2012 | 167 | 71.20 | 0.54 | 40.89 | 28 |
| 15 | Xiaofei Li | 2013 | 1559 | 67.45 | ？ | ？ | 96.2 |
| 16 | Zibin Song | 2016 | 324 | 72.29 | 0.84 | ？ | 48 |
| 17 | Fei Zhou | 2017 | 169 | 75.00 | 0.849 | 33 | 29.6 |
| 18 | Hui-Hsien Hsieh | 2018 | 396 | 68.40 | 0.47 | 33.33 | 65.9 |
| 19 | He Ding | 2019 | 178 | 77.40 | 0.3 | 25.59 | 41.6 |
| 20 | Zhen Wang | 2016 | 377 | 69.40 | 0.43 | 29.93 | 54.6 |
| 21 | Guoping Song | 2010 | 342 | 64.77 | ？ | 27.5 | ？ |
| 22 | Cai-Hui Veronica Lin | 2011 | 423 | 66.00 | 0.38 | 32.62 | 48 |
| 23 | Peizhen Sun | 2016 | 250 | 62.95 | ？ | 27.3 | ？ |
| 24 | Xiaofei Yan | 2015 | 356 | 69.80 | 0.48 | 29.41 | 100 |
| 25 | Xiaofei Yan | 2015 | 524 | 68.20 | 0.45 | ？ | 100 |
| 26 | Zhining Wang | 2018 | 498 | 61.68 | 0.487 | ？ | ？ |
| 27 | Huaiyong Wang | 2017 | 254 | 55.00 |  | 35.04 | 100 |
| 28 | Hongping Zhang | 2012 | 235 | 67.60 | 0.56 | 33.33 | 33.33 |
| 29 | He Ding | 2019 | 314 | 69.80 | 0.51 | 35.17 | 53.5 |
| 30 | He Ding | 2019 | 189 | 78.20 | 0.29 | ？ | 44.4 |
| 31 | Rebecca Garden | 2015 | 213 | 67.71 | 0.7 | 25 | 42.14 |
| 32 | Jiaxi Zhang | 2011 | 310 | 64.40 | 0.54 | 19.84 | 0 |
| 33 | Li Wei | 2012 | 726 | 57.71 | 0.67 | 35 | ？ |
| 34 | Wang Zhen | 2010 | 150 | 70.83 | 0.5 | 33 | 15 |
| 35 | Lin Yi | 2011 | 340 | 65.97 | ？ | 28.3 | 63 |
| 36 | Liu Guanghui | 2012 | 592 | 36.86 | ？ | ？ | 45 |
| 37 | Zhang Linlin | 2011 | 447 | 44.47 | ？ | ？ | 50 |
| 38 | Xu Qin | 2014 | 220 | 64.58 | 0.381 | 28 | 58 |
| 39 | Zhen Xiaoming | 2015 | 555 | 75.40 | 0.51 | 31.5 | 63.4 |
| 40 | Zhang Guiping | 2013 | 344 | 66.80 | 0.95 | 37.5 | 41 |
| 41 | Wang Zhen | 2014 | 192 | 68.40 | 0.51 | ? | 51 |
| 42 | Gu Ping | 2015 | 350 | 55.48 | 0.691 | ? | 42 |
| 43 | Sun Lan | 2012 | 245 | 68.58 | 0.574 | 28.6 | 63 |
| 44 | Guo Yixin | 2017 | 551 | 60.00 | 0.46 | ? | 44 |
| 45 | Su Weilin | 2017 | 243 | 63.58 | 0.495 | ? | ? |
| 46 | Jiang Yunjie | 2017 | 689 | 74.60 | 0.56 | ? | 46 |
| 47 | Yuan Lin | 2018 | 424 | 71.00 | 0.79 | ? | 51.4 |
| 48 | Yuan Lin | 2019 | 489 | 67.89 | 0.834 | ? | 56.6 |
| 49 | Sun Jianmin | 2016 | 204 | 66.20 | 0.73 | 26.69 | 62.3 |
| 50 | Chen Bo | 2018 | 388 | 62.81 | 1.438 | ？ | 64.7 |
|  |  |  |  |  |  |  |  |

Notes：name = name of the first author; year = year of data collection; n = sample size; CSE Score is showed drawing on a hundred mark system; age = sample mean age; sex = sex ratio of sample(% female).

Table S2 Year of data collection, GDP per capita, and Unemployment rate in China

| Year | GDP per capita (RMB) | Unemployment rate(%) |
| --- | --- | --- |
| 2010 | 30808 | 4.1 |
| 2011 | 36277 | 4.1 |
| 2012 | 39771 | 4.1 |
| 2013 | 43497 | 4 |
| 2014 | 46912 | 4.1 |
| 2015 | 49922 | 4 |
| 2016 | 53783 | 4 |
| 2017 | 59592 | 3.9 |
| 2018 | 65534 | 4 |
| 2019 | 70328 | 3.6 |

Notes： Data from National Bureau of Statistics of China (https://data.stats.gov.cn/easyquery.htm?cn=C01).

1 RMB = 0.1544 United States dollar（USD）（08-25-2021）

**A paper list of our meta-analysis**

Aryee, S., Walumbwa, F. O., Mondejar, R., & Chu, C. W. L. (2016). Core Self-Evaluations and Employee Voice Behavior. *Journal of Management, 43*(3), 946-966. doi:10.1177/0149206314546192

Cheung, Y. H., Herndon, N. C., & Dougherty, T. W. (2015). Core self-evaluations and salary attainment: the moderating role of the developmental network. *The International Journal of Human Resource Management, 27*(1), 67-87. doi:10.1080/09585192.2015.1042897

Ding, H., & Lin, X. (2019). Can core self-evaluations promote employee strengths use? *Journal of Psychology in Africa, 29*(6), 576-581. doi:10.1080/14330237.2019.1691792

Ding, H., & Lin, X. (2020). Individual-focused transformational leadership and employee strengths use: the roles of positive affect and core self-evaluation. *Personnel Review, 50*(3), 1022-1037. doi:10.1108/pr-10-2019-0541

Ding, H., & Yu, E. (2020). Follower Strengths-based Leadership and Follower Innovative Behavior: The Roles of Core Self-evaluations and Psychological Well-being. *Revista de Psicología del Trabajo y de las Organizaciones, 36*(2), 103-110. doi:10.5093/jwop2020a8

Ding, H., Yu, E., & Li, Y. (2020). Transformational leadership and core self-evaluation: The roles of psychological well-being and supervisor-subordinate guanxi. *Journal of Psychology in Africa, 30*(3), 236-242. doi:10.1080/14330237.2020.1767934

Garden, R., Hu, X., Zhan, Y., & Yao, X. (2017). Popularity Procurement and Pay Off: Antecedents and Consequences of Popularity in the Workplace. *Journal of Business and Psychology, 33*(2), 297-310. doi:10.1007/s10869-017-9494-9

Hsieh, H.-H., Wang, Y.-C., & Huang, J.-T. (2019). Core self-evaluations, perceived organizational support, and work-related well-being. *Personnel Review, 48*(3), 659-671. doi:10.1108/pr-04-2018-0128

Hsieh, H. H., & Huang, J. T. (2017). Core Self-Evaluations and Job and Life Satisfaction: The Mediating and Moderated Mediating Role of Job Insecurity. *J Psychol, 151*(3), 282-298. doi:10.1080/00223980.2016.1270888

Hu, J., Wang, Z., Liden, R. C., & Sun, J. (2012). The influence of leader core self-evaluation on follower reports of transformational leadership. *The Leadership Quarterly, 23*(5), 860-868. doi:10.1016/j.leaqua.2012.05.004

Hu, Y., Wang, M., Kwan, H. K., & Yi, J. (2019). Mentorship quality and mentors’ work-to-family positive spillover: the mediating role of personal skill development and the moderating role of core self-evaluation. *The International Journal of Human Resource Management, 32*(9), 1899-1922. doi:10.1080/09585192.2019.1579244

Kong, F., Wang, X., & Zhao, J. (2014). Dispositional mindfulness and life satisfaction: The role of core self-evaluations. *Personality and Individual Differences, 56*, 165-169. doi:10.1016/j.paid.2013.09.002

Li, X., Guan, L., Chang, H., & Zhang, B. (2014). Core self-evaluation and burnout among Nurses: the mediating role of coping styles. *PLoS One, 9*(12), e115799. doi:10.1371/journal.pone.0115799

Liang, J., & Gong, Y. (2013). Capitalizing on proactivity for informal mentoring received during early career: The moderating role of core self‐evaluations. *Journal of Organizational Behavior, 34*(8), 1182-1201.

Lin, C.-H. V., & Sun, J.-M. J. (2018). Chinese employees’ leadership preferences and the relationship with power distance orientation and core self-evaluation. *Frontiers of Business Research in China, 12*(1). doi:10.1186/s11782-018-0027-9

O'Neill, T. A., McLarnon, M. J. W., Xiu, L., & Law, S. J. (2016). Core self-evaluations, perceptions of group potency, and job performance: The moderating role of individualism and collectivism cultural profiles. *Journal of Occupational and Organizational Psychology, 89*(3), 447-473. doi:10.1111/joop.12135

Peng, Y.-C., Chen, L.-J., Chang, C.-C., & Zhuang, W.-L. (2016). Workplace bullying and workplace deviance. *Employee Relations, 38*(5), 755-769. doi:10.1108/er-01-2016-0014

Rode, J. C., Judge, T. A., & Sun, J.-M. (2012). Incremental Validity of Core Self-Evaluations in the Presence of Other Self-Concept Traits. *Journal of Leadership & Organizational Studies, 19*(3), 326-340. doi:10.1177/1548051812442964

Song, G., Kong, F., & Jin, W. (2012). Mediating Effects of Core Self-Evaluations on The Relationship Between Social Support and Life Satisfaction. *Social Indicators Research, 114*(3), 1161-1169. doi:10.1007/s11205-012-0195-5

Song, Z., Chon, K., Ding, G., & Gu, C. (2015). Impact of organizational socialization tactics on newcomer job satisfaction and engagement: Core self-evaluations as moderators. *International Journal of Hospitality Management, 46*, 180-189. doi:10.1016/j.ijhm.2015.02.006

Song, Z., Pratt, S., & Wang, Y. (2017). Core self‐evaluations and residents' support for tourism: Perceived tourism impacts as mediators. *International Journal of Tourism Research, 19*(3), 278-288.

Sun, P., & Jiang, H. (2016). Psychometric Properties of the Chinese Version of Core Self-Evaluations Scale. *Current Psychology, 36*(2), 297-303. doi:10.1007/s12144-016-9418-2

Usman, M., Liu, Y., Li, H., Zhang, J., Ghani, U., & Gul, H. (2020). Enabling the engine of workplace thriving through servant leadership: The moderating role of core self-evaluations. *Journal of Management & Organization*, 1-19. doi:10.1017/jmo.2020.11

Wang, H., & Li, Y. (2019). Role overload and Chinese nurses’ satisfaction with work-family balance: The role of negative emotions and core self-evaluations. *Current Psychology*. doi:10.1007/s12144-019-00494-5

Wang, Z., Bu, X., & Cai, S. (2018). Core self-evaluation, individual intellectual capital and employee creativity. *Current Psychology, 40*(3), 1203-1217. doi:10.1007/s12144-018-0046-x

Wang, Z., Xing, L., & Zhang, Y. (2019). Do high-performance work systems harm employees’ health? An investigation of service-oriented HPWS in the Chinese healthcare sector. *The International Journal of Human Resource Management, 32*(10), 2264-2297. doi:10.1080/09585192.2019.1579254

Wang, Z., & Xu, H. (2017). When and for Whom Ethical Leadership is More Effective in Eliciting Work Meaningfulness and Positive Attitudes: The Moderating Roles of Core Self-Evaluation and Perceived Organizational Support. *Journal of Business Ethics, 156*(4), 919-940. doi:10.1007/s10551-017-3563-x

Yan, X., Su, J., Wen, Z., & Luo, Z. (2017). The Role of Work Engagement on the Relationship Between Personality and Job Satisfaction in Chinese Nurses. *Current Psychology, 38*(3), 873-878. doi:10.1007/s12144-017-9667-8

Yan, X., Wang, Z., Su, J., & Luo, Z. (2017). Relationship between core self-evaluations and team identification: The perception of abusive supervision and work engagement. *Current Psychology, 39*(1), 121-127. doi:10.1007/s12144-017-9749-7

Zhang, H., Kwan, H. K., Zhang, X., & Wu, L.-Z. (2012). High Core Self-Evaluators Maintain Creativity. *Journal of Management, 40*(4), 1151-1174. doi:10.1177/0149206312460681

Zhang, J., Wu, Q., Miao, D., Yan, X., & Peng, J. (2013). The Impact of Core Self-evaluations on Job Satisfaction: The Mediator Role of Career Commitment. *Social Indicators Research, 116*(3), 809-822. doi:10.1007/s11205-013-0328-5

Zhou, F., & Wu, Y. J. (2018). How humble leadership fosters employee innovation behavior. *Leadership & Organization Development Journal, 39*(3), 375-387. doi:10.1108/lodj-07-2017-0181
